# A patient-derived missense mouse model of Kabuki syndrome 1

**DOI:** 10.1242/dmm.052775

**Published:** 2026-10-14

**Authors:** Sara Tholl Halldorsdottir, Meghna Vinod, Hilmar Orn Gunnlaugsson, Ellen Dagmar Bjornsdottir, Romain Maurice Jacques Lasseur, Teresa Luperchio, Jill A. Fahrner, Agnes Ulfig, Hans Tomas Bjornsson

**Affiliations:** ^1^ The Louma G. Laboratory of Epigenetic Research, Faculty of Medicine, University of Iceland, Reykjavik 102, Iceland; ^2^ Department of Genetics and Molecular Medicine, Landspitali University Hospital, Reykjavik 101, Iceland; ^3^ McKusick-Nathans Department of Genetic Medicine, Johns Hopkins University School of Medicine, Baltimore, MD 21205, USA; ^4^ Department of Pediatrics, Johns Hopkins University, Baltimore, MD 21205, USA

**Keywords:** KMT2D, Histone machinery, Epigenetics, Mouse model

## Abstract

Kabuki syndrome type 1 (KS1) is a rare cause of intellectual disability resulting from heterozygous pathogenic variants in the gene encoding the histone methyltransferase KMT2D. A previously established loss-of-function mouse model of KS1 exhibits key phenotypic features, and therapeutic trials in this mouse model suggest postnatal malleability of neurological symptoms. However, 15-30% of individuals with KS1 carry missense variants. To investigate whether missense variants lead to similar phenotypic presentation in mice, we used CRISPR-Cas9 to introduce the KS1 patient variant R5179H, corresponding to R5230H in mice, into C57BL/6NTac animals. Computational and *in vitro* testing suggested that the R5230H variant does not impair protein stability or enzyme function of KMT2D. Despite a distinct mechanistic basis, our new mouse model (*Kmt2d^+/R5230H^*) recapitulated most phenotypes of our prior loss-of-function model, including growth deficiency, craniofacial anomalies and IgA deficiency, but not altered neurological function. *Kmt2d^+/R5230H^* mice showed perinatal lethality and a high frequency of unilateral kidney agenesis, a novel phenotype in KS1 mouse models. *Kmt2d^+/R5230H^* mice provide a unique opportunity to understand the impact of missense variants on KMT2D function and uncover developmental and perinatal abnormalities in KS1.

## Teaser

**Summary:** A new missense mouse model of Kabuki syndrome 1 (KS1) with intact KMT2D enzymatic function has most features of prior KS1 models, except disruption of adult neurogenesis, and exhibits unilateral renal agenesis.

## INTRODUCTION

Extensive work has been conducted over the past 10 years on therapeutic development for Kabuki syndrome type 1 (KS1) using the *Kmt2d^+/βGeo^* mouse model, which recapitulates many defining features of individuals with KS1 (Comel et al., 2025; Harris et al., 2019; Ruault et al., 2020; Schott et al., 2017), resulting in several promising therapeutic studies (Benjamin et al., 2017; Bjornsson et al., 2014; Zhang et al., 2021). Observed features in the mice include growth deficiency with flattened facial profile (Bjornsson et al., 2014; Fahrner et al., 2019), a visuospatial learning defect (Bjornsson et al., 2014) and immunoglobulin A (IgA) deficiency (Pilarowski et al., 2020). Although this model is thought to have a similar loss-of-function mutational mechanism to the majority of individuals with KS1 (Hannibal et al., 2011), it was generated by inserting an artificial β-galactosidase neomycin cassette (*Kmt2d^+/ßgeo^*), which, theoretically, could have additional effects on the function of the histone-lysine N-methyltransferase 2D (KMT2D) protein, such as altered structure, resulting in aberrant binding. Moreover, this only reflects a single loss-of-function allele, and the molecular cause for ∼15-30% of individuals with KS1 involves heterozygous missense variants (Cocciadiferro et al., 2018; Hannibal et al., 2011), highlighting that this model is not representative of the full molecular spectrum of KS1. These KS1-causing missense variants have been shown to be enriched at both the enzymatic (SET) and the FYRN domains (Faundes et al., 2019). In contrast, missense variants in KMT2A, another H3K4 methyltransferase that causes Wiedemann–Steiner syndrome, shows enrichment at the CpG binding domain (CXXC) but not the enzymatic SET domain (Reynisdottir et al., 2022). The observation that pathogenic missense variants in these related syndromes preferentially cluster within protein interaction and DNA-binding domains (FYRN and CXXC), rather than consistently within the catalytic SET domain, suggests that disruption of the binding and regulatory functions of the related methyltransferases KMT2A and KMT2D may be important in disease pathogenesis.

Multiple treatment strategies countering the observed epigenetic abnormalities have shown promise in the *Kmt2d^+/ßgeo^* mouse model, rescuing the neurological deficits in postnatal life (Benjamin et al., 2017; Bjornsson et al., 2014; Zhang et al., 2021). However, it remains unclear whether the same treatments would also work in mouse models with a different mutational mechanism. One prior model harboring a missense variant exists, which is associated with a KS1-like phenotype (Yamamoto et al., 2019). This missense variant lies within exon 13 and functions as a hypomorph, with phenotypes only observed in homozygous animals, which does not reflect what is seen in individuals with KS1. Thus, establishing a patient-relevant mouse model of KS1 may offer novel insights into the pathophysiology of this disease.

Here, we used CRISPR-Cas9 to introduce a patient-derived variant into the corresponding sequence of murine *Kmt2d*. Although protein expression and enzyme function of KMT2D was not affected, *Kmt2d^+/R5230H^* mice had extensive perinatal lethality and many overlapping phenotypes with the prior model (Bjornsson et al., 2014), including craniofacial defect, growth deficiency, IgA deficiency and fewer Peyer's patches. However, the phenotypes of altered neurological function, visuospatial learning defects and decreased global histone methylation levels seen in the prior model were not found in these mice. In addition, unilateral kidney agenesis emerged as a novel phenotype that has not been previously observed in KS1 mouse models. We believe that this model will be useful for the KS1 community to aid in the investigation of the mechanistic basis of KS1 and to explore previous and novel therapeutic modalities for KS1.

## RESULTS

### High mortality in a mouse model with a patient-derived missense variant in the FYRN domain

In an effort to extend the availability of representative KS1 mouse models, we aimed to generate a mouse model with a patient-specific missense variant. We examined the list of patients with missense variants seen in our Epigenetics and Chromatin Clinic at Johns Hopkins Hospital. We selected a variant (c.G15536A:p.R5179H) located within the FYRN domain and seen in one of our patients with classical symptoms of KS1 and a score comparable to that in patients with loss-of-function variants (Table 1). Mice carrying the corresponding variant c.15689_90GA>AC:p.R5230H (*Kmt2d^+/R5230H^*) were generated using CRISPR-Cas9 and subsequently subjected to phenotyping. The corresponding mouse variant involves a change from an arginine (R) to a histidine (H) at a conserved region within the FYRN domain (Fig. 1A,B). CRISPR-Cas9 off-target effects were excluded at locations predicted to have three or fewer mismatches. The predicted sites were at the *Samd8* and *Cd163* loci screened in F1 animals and F4 offspring (Fig. S1A). Additionally, the mouse phenotypes were examined throughout five generations because a second variant would be expected to randomly segregate off and, therefore, be unlikely to be the cause of the observed phenotypes. We used AlphaFold3 to model the potential structure of the FYRN/FYRC domain and the change in structure upon introduction of R5230H. AlphaFold predicted (>70% confidence) that this variant localizes within a β-sheet and that the change is not expected to alter the gross structure of KMT2D (Fig. 1C); however, the changes in side-chain structure are likely to disrupt hydrogen bonding (Fig. 1D), potentially affecting the local interaction networks. Additionally, visualization of the predicted electrostatic potential of this domain displayed a change to a less positive charge (Fig. 1E). To investigate potential interactors of this region, we performed protein–protein prediction against components of the COMPASS complex (ASH2L, DPY30, KMT2C, NCOA6, PAXI1-PGR1A, RBBP5, UTX and WDR5) using a part of the C-terminal region of KMT2D. Aside from WDR5, which is known to bind to the WIN motif at this region, no other interactions were predicted with high confidence (interface predicted template modeling score>0.8). However, when visualized, two predictions resulted in a probable interaction shown with connectors to the specific region of the variant, NCOA6 (Fig. S1B) and/or the PAXI1-PGR1A partners (Fig. S1D). In both instances, this interaction was predicted to be present at the amino acid R5230 and not predicted after the R5230H substitution (Fig. S1C,E). Both factors are known interactors of KMT2D through their participation in the COMPASS complex (Cho et al., 2007; Lee et al., 2006).

**Fig. 1.**
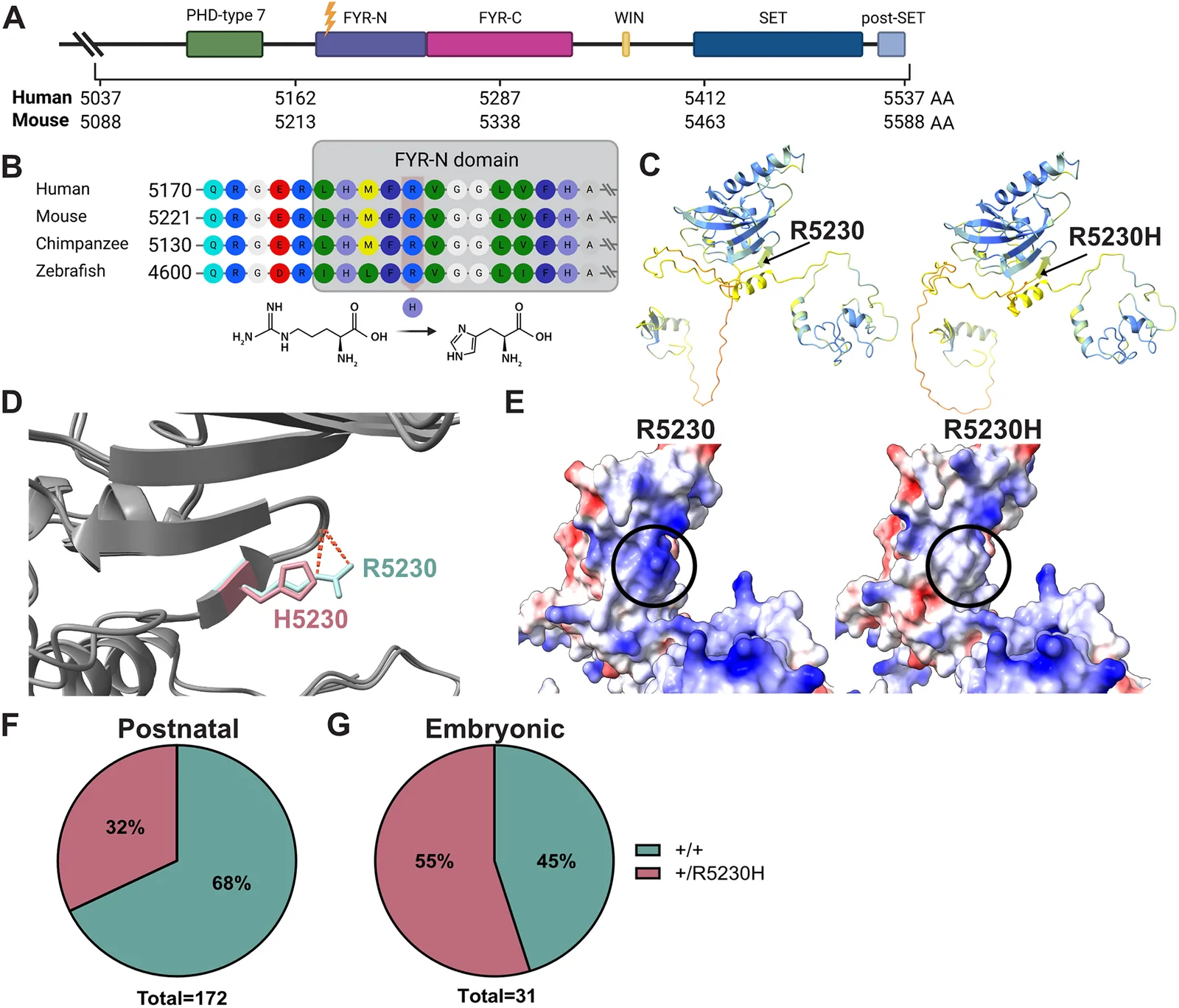
**The R5230H variant interrupts hydrogen bonds and changes the electrostatic charge of the FYRN domain.** (A) A schematic view of the human and mouse KMT2D C-terminus including 500 amino acids, with the variant location marked with a lightning symbol. AA, amino acids. (B) Partial amino acid sequences around the missense variant in various organisms with the R5230H substitution highlighted. The molecular structures of arginine and histidine are shown below. Amino acids are colored according to the RasMol amino acids color scheme. (C) AlphaFold3 prediction for the FYRN/FYRC domains, with 100 flanking amino acids colored by pLDDT, with orange representing the least confidence and dark blue representing the highest confidence. (D) Amino acid side chains for R5230 (green) and H5230 (pink), with hydrogen bonds shown in red dashed lines. (E) Electrostatic charge of the FYRN/FYRC domains, with amino acid R5230 circled. Blue indicates a positive charge; red indicates a negative charge. (F,G) Mendelian ratios for postnatal (F) and embryonic [embryonic day (E)16.5-18.5; G] survival. ‘Total’ indicates the number of animals used for calculations.

**Table 1.** Symptoms present in a patient with Kabuki syndrome 1 carrying the c.G15536A:p.R5179H variant

Kabuki syndrome 1 features		Patient features	Kabuki score
Facial features (1-5 points; zero to three features, 1 point; four to six features, 2 points; seven to nine features, 3 points; ten to 12 features, 4 points; 13 to 15 features, 5 points)	Arched eyebrows with sparse lateral third	×	3
	Long palpebral fissures	×	
	Everted lower eyelids	×	
	Ptosis	−	
	Strabismus	×	
	Blue sclerae	−	
	Large dysplastic ears	×	
	Broad nasal root	−	
	Flat nasal tip	×	
	Abnormal dentition	×	
	Oligodontia	×	
	High or cleft palate	×	
	Micrognathia	−	
	Thin vermilion of the upper lip and full lower lip	−	
	Lip nodules	−	
Limb and extremity features (0-1 point; zero to one feature, 0 points; two to four features, 1 point)	Brachydactyly or clinodactyly	×	1
	Hip dislocation	−	
	Lax joints	×	
	Persistent fetal pads	×	
Microcephaly (1 point)		−	−
Short stature (1 point)		×	1
Heart (1 point)		−	−
Kidney (1 point)		−	−
Total (0-10 points)			5

We observed homozygous lethality of mice carrying the variant. In contrast to the previous model, however, in the heterozygous state, the Mendelian ratio was much more skewed (Bjornsson et al., 2014), with only ∼30% live-born heterozygotes (50% expected) (Fig. 1F). However, this number did not consider cohorts with no live-born heterozygotes, indicating even higher perinatal lethality of *Kmt2d^+/R5230H^*. To investigate whether the lethality occurred during the embryonic period, we performed timed breeding and sacrificed dams at embryonic day (E)16.5-18.5. In those cases, we observed a Mendelian ratio closer to that expected with 55% of the embryos carrying the R5230H variant (Fig. 1G). This suggested that lethality occurs perinatally rather than during embryonic life.

### The R5230H variant does not alter the expression or enzymatic function of KMT2D

Gene expression studies on hippocampal tissue showed that the R5230H variant does not affect stability of *Kmt2d* mRNA, as evident by unaltered *Kmt2d* mRNA levels between *Kmt2d^+/R5230H^* and *Kmt2d^+/+^* mice when amplifying either regions upstream (exons 2-3 and 3-4) or downstream (exons 49-50 and 51-52) of the variant (Fig. 2A). Similarly, western blots for KMT2D in spleen tissue (Fig. 2B) and mesenchymal stem cells (Fig. S2A) did not show any significant differences in protein levels between samples derived from *Kmt2d^+/R5230H^* and *Kmt2d^+/+^* animals. When testing the global enzymatic activity by quantifying H3K4me1 and H3K4me3 levels with western blotting, we did not observe decreased H3K4me1 or H3K4me3 levels in *Kmt2d^+/R5230H^* compared to *Kmt2d^+/+^* mice (Fig. 2C,D). Together, these results suggest that the R5230H variant does not affect KMT2D protein expression or cause a drastic loss of KMT2D enzymatic function.

**Fig. 2.**
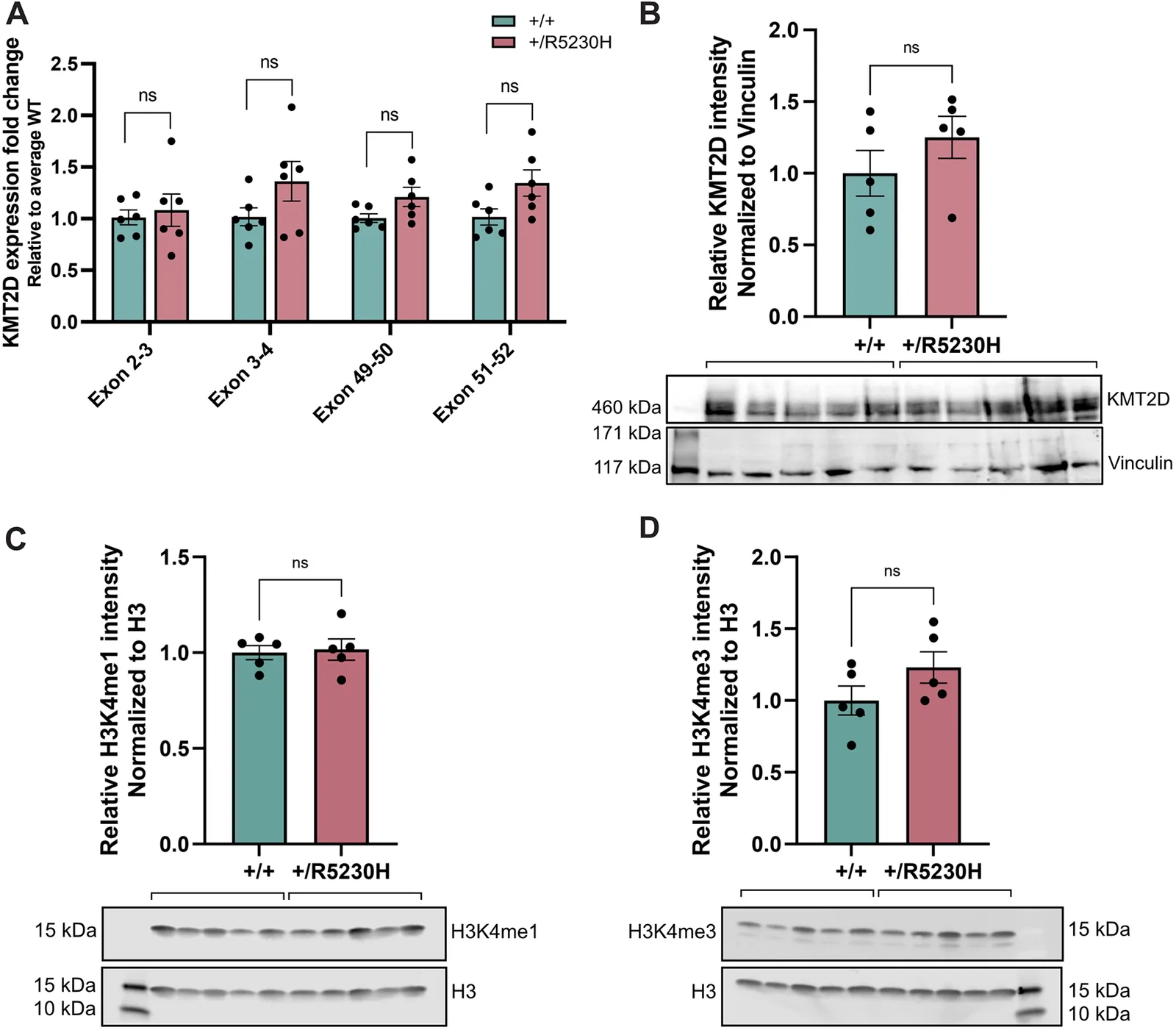
**R5230H does not cause changes in KMT2D expression or global methylation.** (A) Relative mRNA expression of *Kmt*2*d* from hippocampal tissue detected with four primer pairs (exons 2-3, 3-4, 49-50 or 51-52) using the geometric mean of *Actb* and *Hprt* for normalization. (B-D) Western blots and quantification of KMT2D with vinculin as control (B), and H3K4me1 (C) or H3K4me3 (D) using H3 as control, with protein isolated from spleen tissue. Both males and females were used (*n*=5-6 per genotype). Data are represented as mean±s.e.m. Unpaired two-tailed Student's *t*-tests were used for statistical analysis. ns, not significant.

### *Kmt2d^+/R5230H^* mice show core phenotypes of KS1 except disruption of adult neurogenesis

*Kmt2d^+/R5230H^* mice showed growth deficiency, with significantly decreased body weight compared to that of *Kmt2d^+/+^* mice (*P*<0.0001; Fig. 3A). To determine whether the R5230H variant produced craniofacial alterations similar to those observed in a previously described mouse model (Fahrner et al., 2019), we performed micro-computed tomography (microCT) imaging (Fig. 3B) and used 16 anatomical landmarks for morphometric analyses (Fig. 3C). Independent Procrustes ANOVA revealed that nine of the 16 landmarks were displaced in *Kmt2d^+/R5230H^* compared to *Kmt2d^+/+^* mice (Fig. 3D; Fig. S2B), identifying anatomical drivers of craniofacial shape variation. Principal component analysis (PCA) demonstrated clear separation between genotypes based on craniofacial shape (Fig. 3E), indicating significant morphological changes. Regression analyses examining the relationship between shape and size revealed similar allometric slopes between genotypes, while maintaining distinct trajectory separation, showing that the observed craniofacial differences were largely shape specific rather than driven by size alone (Fig. 3F). Cross-validation of the discriminant analysis derived from the first five principal components yielded an overall classification accuracy of 93.75%, demonstrating clear separation between *Kmt2d^+/R5230H^* and *Kmt2d^+/+^* mice, suggesting a significant and quantifiable morphological impact of the R5230H missense variant. Notably, all animals clustered according to genotype except for a single mutant outlier that localized within the wild-type space (Fig. S2C). Other features that resembled the previous model were the significantly decreased body length (*P*<0.05; Fig. S2D), decreased long bone length (femur, *P*<0.01; tibia, *P*<0.05; Fig. 3G), lower IgA blood serum levels (*P*<0.0001; Fig. 3H) and reduced number of Peyer's patches (*P*<0.01; Fig. 3I) compared to those in *Kmt2d^+/+^* mice. These results suggested that these phenotypes are not dependent on the type of variant or secondary to the βGeo tag in the prior model (Bjornsson et al., 2014). Behavioral testing showed that *Kmt2d^+/R5230H^* mice exhibited no significant difference in open field testing (Fig. S2E) or distance traveled in a Morris water maze (Fig. 4A) compared to *Kmt2d^+/+^* mice, indicating that they do not display anxiety-like behavior. To test visuospatial learning, we performed a Morris water maze platform test. *Kmt2d^+/R5230H^* mice showed no significant difference in platform crossings (Fig. 4B) or proportion of time spent in the platform quadrant (Fig. 4C) compared to *Kmt2d^+/+^* littermates. Additionally, visible platform testing showed that both *Kmt2d^+/+^* and *Kmt2d^+/R5230H^* mice had the ability to learn across training days with significant effect of time (two-way repeated-measures ANOVA; *P*<0.0001) and no significant difference observed in escape latency between genotypes (*P*=0.172). However, a significant time and genotype interaction was observed (*P*=0.0457), suggesting that *Kmt2d^+/R5230H^* mice showed a slightly different learning pattern to *Kmt2d^+/+^* mice (Fig. S2F). To further test the visuospatial memory of these mice, we also performed a Y-maze test. Similar to the Morris water maze test, this behavior test showed no significant difference in novel arm entry (Fig. 4D), time spent in the novel arm (Fig. 4E) or spontaneous alternations (Fig. 4F) in *Kmt2d^+/R5230H^* compared to *Kmt2d^+/+^* animals. To further validate unaltered neurodevelopment at a cellular level, we stained cryosectioned brains and examined the region of the dentate gyrus (Fig. 4G). We did not observe reduced volume of the dentate gyrus granule cell layer (Fig. 4H) or a difference in numbers of 5-ethynyl-2′-deoxyuridine (EdU)-stained cells in the hippocampus (Fig. 4I), suggesting relatively normal proliferation and differentiation of neurons of the hippocampus. Additionally, staining of the hippocampus for H3K4me3 did not reveal decreased methylation levels (Fig. 4J,K). This supports what we previously described for spleen tissue (Fig. 2D), i.e. unchanged global methylation levels upon the introduction of the R5230H variant. Together, these findings suggest that our novel missense mouse model replicates the majority of core disease phenotypes seen in individuals with KS1, except features related to disruption of adult neurogenesis.

**Fig. 3.**
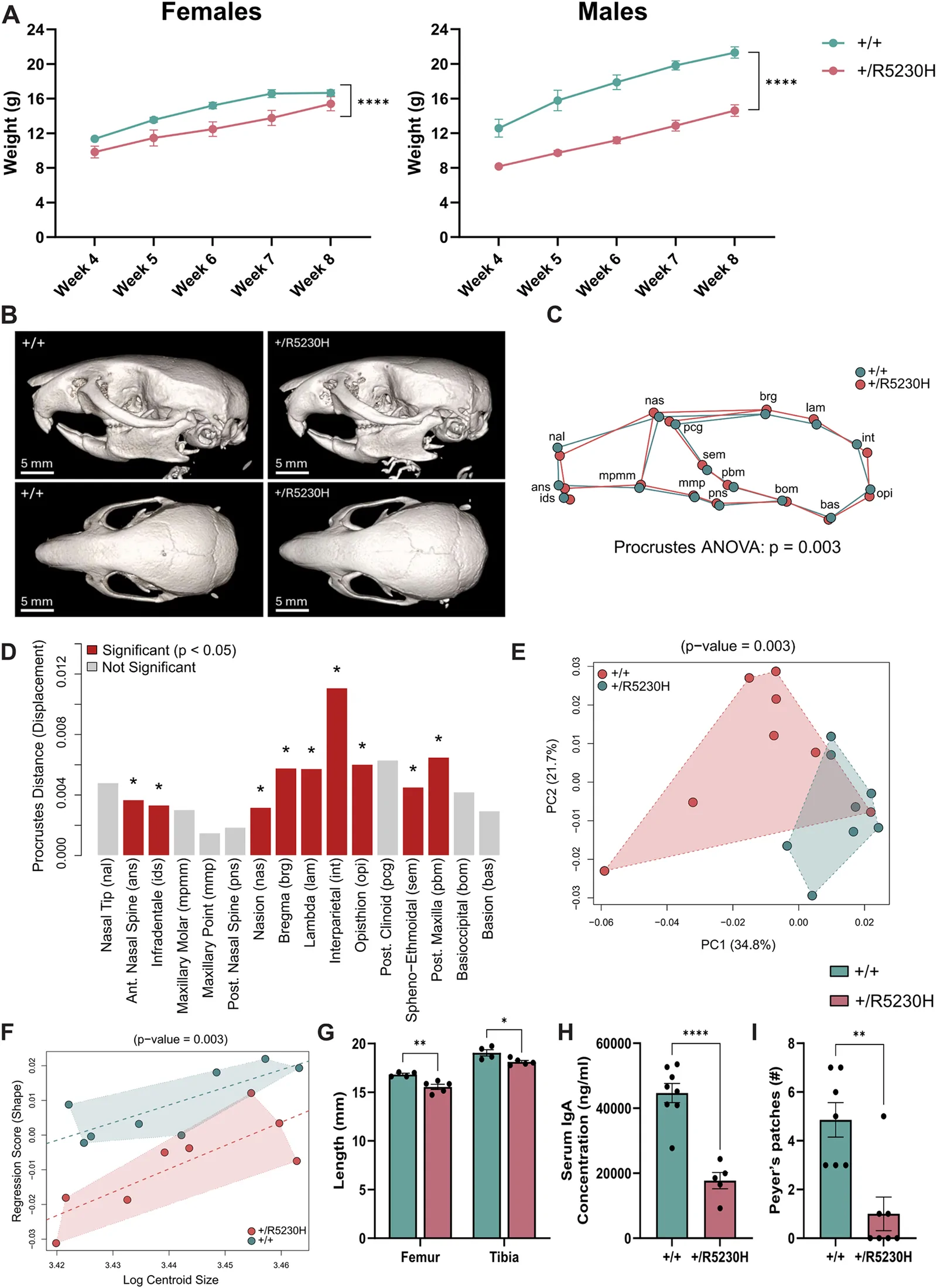
**Growth, craniofacial, skeletal and immune phenotypes in the *Kmt*2*d^+/R^^5230^^H^* mouse model.** (A) Body weights of female (left, *n*=6-8) and male (right, *n*=6-13) *Kmt*2*d^+/+^* and *Kmt*2*d^+/R5230H^* mice. (B) Representative 3D micro-computed tomography reconstructions of the lateral (top row) or top (bottom row) views of *Kmt*2*d^+/+^* (left column) or *Kmt*2*d^+/R^^5230^^H^* (right column) mice. (C) Overlaid Procrustes shape comparison between *Kmt*2*d^+/+^* (green) and *Kmt*2*d^+/R^^5230^^H^* (red) mice, illustrating the morphological differences between the groups. (D) Quantitative analysis of landmark displacement along the craniofacial axis. Landmarks showing significant displacement in the *Kmt*2*d^+/R^^5230^^H^* mice are displayed in red. (E) Principal component analysis plot of the craniofacial features, showing a difference along PC1 (*n*=7). PC, principal component. (F) Scatter plot illustrating the allometric relationship between size (log-transformed centroid size) and shape (regression score) for the two genotypes. The dashed lines represent the linear regression for each group, highlighting that the specific allometric trajectory (how shape changes as size increases) for each genotype follows a similar allometric pattern. (G) Femur and tibia measurements of 3- to 4-month-old mice (in mm; *n*=4-5). (H) Levels of IgA in blood serum quantified at 3 months of age. One outlier was detected among the *Kmt*2*d^+/R^^5230^^H^* (*n*=6) using Grubbs' test (*P*<0.05) and was removed prior to analysis. The final sample sizes were as follows: *Kmt*2*d^+/R^^5230^^H^*, *n*=5; *Kmt*2*d^+/+^*, *n*=8. (I) Number of Peyer's patches quantified by counting (*n*=7). Data are represented as mean±s.e.m. Statistical tests performed were mixed-effects ANOVA (A), Procrustes ANOVA (B-F) and unpaired two-tailed Student's *t*-tests (G-I). *P≤0.05, **P≤0.01, ****P≤0.0001.

**Fig. 4.**
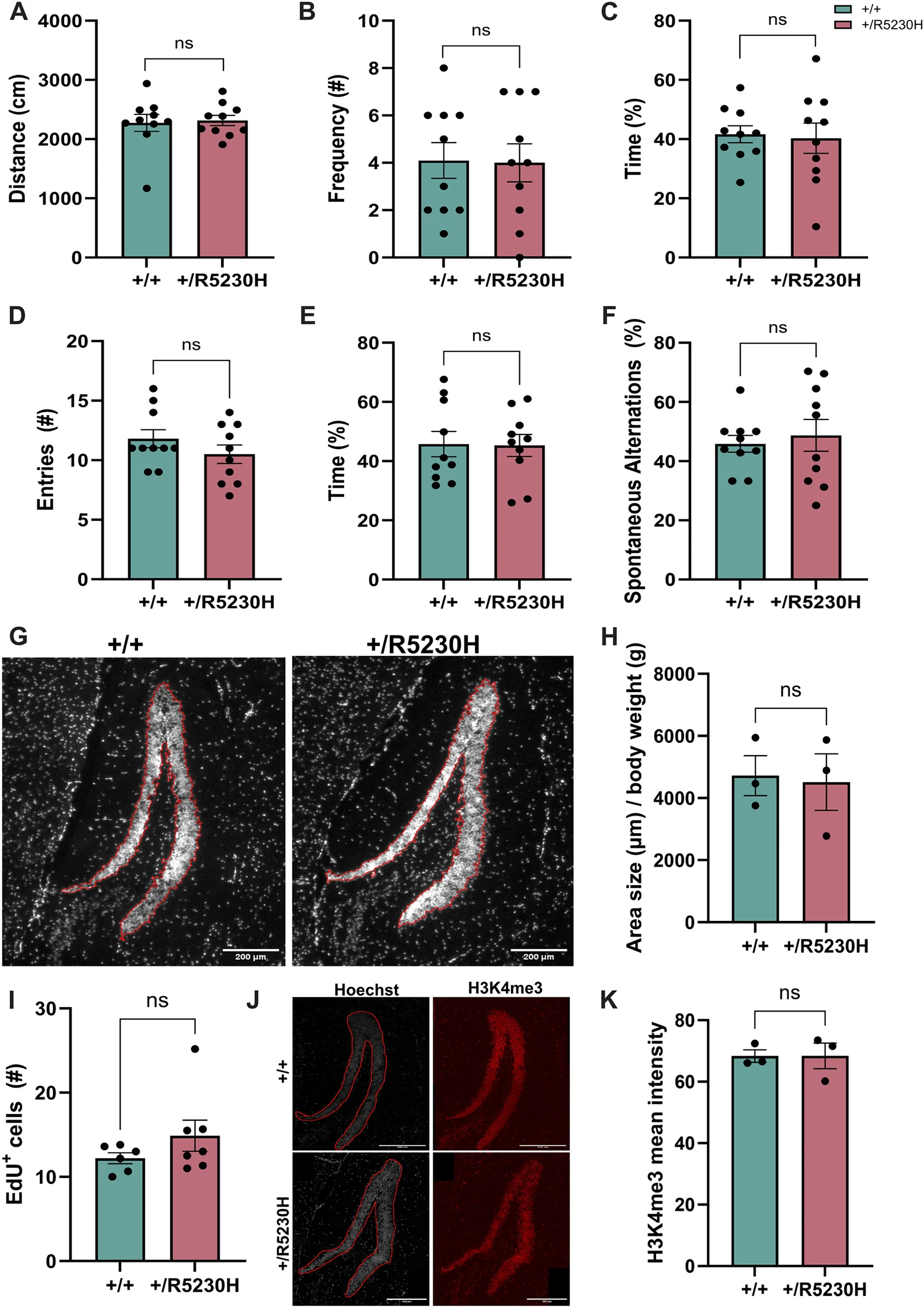
**Behavioral and hippocampal phenotypes in *Kmt*2*d^+/R5230H^* mice.** (A-C) Morris water maze analysis measuring total distance traveled (A), total platform crossings (B) and percentage of time spent in the platform quadrant relative to total time spent in maze (C). (D-F) Y-maze analysis measuring the number of novel arm entries (D), the percentage of time spent in novel arm relative to total time (E) and spontaneous alternations (F). (G,H) Representative images of brain sections stained with Hoechst dye with the granule cell layer outlined (G) and quantification in μm (H). Scale bars: 200 μm. (I) Quantification of number of proliferative 5-ethynyl-2′-deoxyuridine (EdU)^+^ cells within the granule cell layer. (J) Representative images of H3K4me3 staining within the granule cell layer. Hippocampus area measured was outlined with Hoechst staining. Scale bars: 1000 μm. (K) Quantification of H3K4me3 hippocampal mean intensity per area. Unpaired two-tailed Student's *t*-tests were performed; data are represented as mean±s.e.m. This study was limited to females (*n*=10) because males were used for breeding.

In addition to these common phenotypes, we observed a phenotype in *Kmt2d^+/R5230H^* mice not seen in other KS1 mouse models. Approximately 43% of *Kmt2d^+/R5230H^* animals showed unilateral renal agenesis (Fig. S3A). This abnormality was not seen in any of the *Kmt2d^+/+^* animals and, to the best of our knowledge, has not been observed or reported in *Kmt2d^+/βGeo^* mice. Loss of a single kidney often results in enlargement of the remaining kidney (Woolf and Hillman, 2007), prompting us to conduct further histological investigation. Histological staining revealed normal gross histology in *Kmt2d^+/R5230H^* animals with one kidney (Fig. S3C), but size quantification showed enlargement of the glomeruli within the single kidney compared to those in *Kmt2d^+/R5230H^* animals that presented with two kidneys or *Kmt2d^+/+^* animals (Fig. S3B). These results suggest that this particular model could be valuable for studying the impact of KMT2D dysfunction in kidney development and abnormalities.

## DISCUSSION

Here, we describe a novel missense mouse model of KS1 based on a patient-specific missense variant located in the FYRN domain of KMT2D. This variant was selected given that it has been found multiple times in individuals with KS1. One of the individuals that we follow with KS1 carries this variant and has multiple features of KS1 including classic dysmorphic features. In addition, this particular individual exhibited an unusually high IQ (77) compared to other individuals with KS1, as well as a history of neuropsychiatric issues, which are not common in KS1. The FYRN domain is a known hotspot of missense variants specific to individuals with KS1 compared with variants found in the general population or different types of cancer (Faundes et al., 2019). According to ClinVar and Faundes et al. (2019), there are 17 missense variants occurring in 12 amino acids reported within the FYRN domain, all described as being disease causing. The selected patient variant is the arginine residue most frequently substituted in KS1, R5179, and has been observed to be replaced by C, H, L or P – with H recurring in multiple individuals. Similar recurrent substitutions within the FYRN domain have been reported at Arg5214 (Faundes et al., 2019). Functional studies of missense variants in KS1 have been carried out by Cocciadiferro et al. (2018). In that study, two missense variants present in the FYRN domain, G5189R and W5217R, were tested. These variants were predicted to alter the structure of the domain similarly to R5230H, as well as change the total charge. Interestingly, upon introduction of a mini-KMT2D vector spanning the PHD4-5-6 domains and ZF-PHD7-FYRN-FYRC-WIN-SET-post SET domains, these variants both led to decreased catalytic activity as well as decreased interaction with ASH2L/RBBP5 (Cocciadiferro et al., 2018). To our knowledge, other variants within the domain have not been functionally tested. The FY-rich N- and C-terminal sequence motifs form a single folded FYR domain enriched in phenylalanine/tyrosine that is found in many chromatin modifying proteins, specifically common in H3K4 methyltransferases (García-Alai et al., 2010). Limited information exists about the specific function of the FYR domain of KMT2D, but it has been shown that deletion of either part of the domain (FYRN or FYRC) in the demethylase, JMJ14, impairs its chromatin binding capacity rather than affects its catalytic function (Zhang et al., 2015). Even though there are limitations in using AlphaFold for large proteins such as KMT2D, our predictions using ∼400-500 amino acids of the C-terminus are consistent with previous reports, showing that the FYR domain adopts an α+β structure. Our predictions revealed that R5230H is located within a β-sheet that often mediates protein binding (García-Alai et al., 2010; Nowick, 2008) and suggests changes in electrostatic charge at predicted binding sites. *In silico* analysis revealed that the amino acid substitution is predicted to affect protein interaction of known components of the COMPASS complex, NCOA6 and partners PAXI1-PGR1A, proteins that have both previously been implicated in the recruitment specificity of the complex (Cho et al., 2007; Lee et al., 2006). In order to fully conclude that the amino acid substitution hinders the interaction with these components, functional experiments such as co-immunoprecipitation or chromatin immunoprecipitation followed by sequencing (ChIP-seq) will be needed.

The phenotypes of the *Kmt2d^+/R5230H^* mice show concordance with the majority of phenotypes previously described in *Kmt2d^+/βGeo^* mice, including growth deficiency, craniofacial defects, low IgA serum levels and decreased numbers of Peyer's patches. Interestingly, the postnatal severity of the *Kmt2d^+/R5230H^* model appears to be greater than that observed for *Kmt2d^+/βGeo^* mice, as evaluated by the number of surviving heterozygotes. We also observed unilateral kidney agenesis, but previous studies suggest that up to 40% of individuals with KS1 have renal malformations (Banka et al., 2012; Courcet et al., 2013; Hannibal et al., 2011). Such abnormalities have not been described in earlier mouse models of KS1 (Bjornsson et al., 2014), and our findings, therefore, highlight a clinically relevant phenotype that could now be modeled and investigated in mice. The malformations found in individuals with KS1 have a wide range of presentations and severities, which do not appear to correlate with variant type (Banka et al., 2012; Courcet et al., 2013; Hannibal et al., 2011). Similar to observations in our model, a prior case report showed unilateral renal agenesis with renal insufficiency in a patient with a truncating c.9829C>T, p.Gln3277X variant in *KMT2D* (Courcet et al., 2013). The renal agenesis phenotype in *Kmt2d^+/R5230H^* mice was observed in an initial cohort but showed variable penetrance in subsequent cohorts. In addition, the patient carrying the R5179H variant did not present with known renal abnormalities or any renal disease, to the best of our knowledge. We, therefore, emphasize that this is a preliminary observation that will need further validation, as it may reflect incomplete penetrance, strain-specific effects or potential off-target effects related to model generation. Further investigation will be needed to see whether this novel phenotype remains stable or is lost with additional breeding.

Some discrepancies in the observed phenotypes between the two models are noted. No significant difference in global H3K4me3 within the hippocampus was observed in *Kmt2d^+/R5230H^* mice. Moreover, we did not detect changes in hippocampal size or visuospatial learning deficits as seen in the prior model (Bjornsson et al., 2014). Notably, these phenotypes may be secondary to the disruption of adult neurogenesis, leading us to speculate that our model does not exhibit the same extent of neurological dysfunction as the prior model. These findings are supported by the fact that the patient carrying this variant presented with higher-than-expected cognitive abilities (IQ of 77), consistent with mild intellectual disability without any report of visuospatial memory defects. They should, however, be interpreted with caution, as our study may be underpowered to detect subtle effects and was limited to female subjects. Potential sex-specific effects, therefore, cannot be excluded and should be investigated in future studies. It is worth mentioning that these assays might not recapitulate the complete neurological spectrum of KS1, and additional behavioral and electrophysiological experiments are needed. Interestingly, if validated in larger cohorts and with functional studies, these findings raise the possibility that the loss of KMT2D catalytic function is necessary for manifestation of the adult neurogenesis disruption in KS1.

Genotype–phenotype studies have not found a definitive correlation between disease appearance and type of variant (Hannibal et al., 2011; Miyake et al., 2013). A study from 2012, exploring *KMT2D* missense variants, suggests that perhaps not all KS1-causing variants are loss-of-function variants (Banka et al., 2012). Concordantly, another more recent study tested missense variants from individuals with KS1 *in vitro* and found that only ∼65% appeared to reduce H3K4 methyltransferase activity (Cocciadiferro et al., 2018). Furthermore, a new disease entity has recently been described, in which missense variants in exons 38 and 39 of *KMT2D* cause no alterations in histone methylation, and the observed phenotypes are somewhat distinct from KS1. In addition, these individuals did not present with intellectual disability (Cuvertino et al., 2020). Because we do not see changes in H3K4 methylation, this highlights an alternative function for KMT2D and supports the idea that a missense variant could cause changes in a function of KMT2D that are unrelated to its catalytic activity. KMT2D has previously been shown to be important during embryonic development, during which targeted deletion in cardiac precursors causes embryonic lethality (Ang et al., 2016) and whole-body homozygous deletion results in lethality around E9.5 (Lee et al., 2013). Although numerous studies highlight the critical role of KMT2D in development, and some propose a potential mechanistic basis, none have definitively explained its precise function (Froimchuk et al., 2017; Shan et al., 2024). Our findings demonstrate an essential role of KMT2D in development that may be independent of its methyltransferase function.

In summary, here, we present the first patient-specific mouse model of KS1, capturing core KS1 phenotypes along with high perinatal lethality and possible renal agenesis, making it a valuable tool for studying these phenomena in the future. Despite harboring the majority of previously observed KS1 features, it lacks defects in adult neurogenesis and loss of enzyme activity. We, therefore, speculate that the catalytic role of KMT2D might underlie visuospatial defects and neurogenesis disruptions in KS1, but not other traits; however, additional studies will be needed to robustly test this idea.

## MATERIALS AND METHODS

### Animals

#### Mouse model

The mouse model was created by Taconic with a constitutive knock-in of a point mutation via CRISPR-Cas9 with microinjection on C57BL/6NTac zygotes followed by *in vitro* fertilization. The targeting strategy was based on NCBI transcript NM_001033276.3 using gRNA (5′-CGGCTACACATGTTCCGAGTAGG-3′) and homologous recombination donor oligonucleotide containing the knock-in variant. Two founder mice were successfully established, and the line was maintained on pure C57BL/6NTac background. CRISPR-Cas9 off-targets were screened with Sanger sequencing. Polymerase chain reaction (PCR) was performed with primers targeting *Samd8* (5′-GCAGGTGTCGTTCTTATGTTGGGG-3′ and 5′-AGTCAGAGTCATTCCAAAGCCGC-3′) and *Cd163* (5′-TAAGCACTGGGTAGATCACAGG-3′ and 5′-CCCCACTGCCAGTTTTTGCATTGC-3′) using ExtraMix, which was provided by deCODE genetics; Sanger sequencing was performed by deCODE genetics for *Samd8* (5′-ACAGTGACTATGTGAGGCAGGG-3′ or 5′-TTTGTGAGCTTGAGGTCAGCCTGG-3′) and Genewiz for *Cd163* (5′-TAAGCACTGGGTAGATCACAGG-3′ and 5′-CCCCACTGCCAGTTTTTGCATTGC-3′). Genotyping was performed on ear clips with primers R5230H F-(5′-CTTCAAGGACAAGACCATGCTG-3′) and R5230H R-(5′-AGGCAGGGGCATCATTTG-3′) targeting exon and intron 49, flanking the missense variant. The R5230H variant introduces a restriction site, making it possible to cleave the R5230H PCR product with BsaAI restriction enzyme. The following thermal cycler conditions were used for PCR: initial denaturing for 2 min at 96°C followed by 40 cycles of denaturing at 96°C for 30 s, annealing at 57°C for 30 s and extension at 70°C for 1 min. Final extension was performed at 72°C for 10 min.

#### Housing

All animals were bred in a pathogen-free facility at ArcticLAS (Reykjavik, Iceland), under 12-h light/dark cycles at 21-24°C and 40% humidity in cages of six mice maximum. Behavioral testing was done in a University of Iceland Facility (VRIII). Mice were provided with filtered water and kept on separate diets for breeding (Altromin NIH#31M) and maintenance (Altromin 1324) *ad libitum*. Health monitoring was performed regularly under Federation of European Laboratory Animal Science Associations (FELASA) standards, and samples taken were analyzed by IDEXX Germany. Import of all mice and all experimental protocols were approved by the Icelandic Food and Veterinary Authority (license 18081393) and performed in accordance with FELASA guidelines.

### KMT2D variant testing

#### AlphaFold

For protein modeling and prediction, AlphaFold3 was used with the AlphaFold server, developed by Abramson et al. (2024). Input sequences were retrieved from UniProt (Bateman et al., 2017). KMT2D (UniProt ID: Q6PDK2), NCOA6 (UniProt ID: Q9JL19), PAXI1 (UniProt ID: Q6NZQ4), PGR1A (UniProt ID: Q99L02). Predictions were generated using the default parameters, with quality assessed by pLDDT scores. Structural visualization and analysis were conducted using ChimeraX-1.9 (Pettersen et al., 2021).

#### Gene expression

For *Kmt2d* expression, total RNA was extracted from 8-week-old hippocampus with TRIreagent (Thermo Fisher Scientific, AM9738), isolated with Direct-zol RNA MicroPrep kit (Zymo, R2060), and cDNA synthesis was performed with a High-Capacity cDNA Reverse Transcription Kit (Thermo Fisher Scientific, 4368814) according to manufacturer protocols. Real-time quantitative PCR (qPCR) was performed with Luna Universal qPCR Master Mix (NEB, M3003X) on a CFX96 Touch Real-Time PCR Detection System (Bio-Rad) using the following primers: Kmt2d_E51-52_F (5′-AAACGGCCCCATACCTTGAACAGC-3′), Kmt2d_E52_R (5′-TGCACAAACTGCTTGCTGTACGGG-3′), Kmt2d_E49_F (5′-TCAGTGAGAACAATGGACGGCC-3′), Kmt2d_E49-50_R (5′ATGATGCGGTTCCACACAGCTTGG-3′), Kmt2d_E2-3_F (5′-AGATTCAGACCCAGCAGCTGATGG-3′), Kmt2d_E3_R (5′-ACACAGAATGGGAAGGTCTGGC-3′), Kmt2d_E3-4_F (5′-AAGCCTCCTCATGACTGCAGTAGG-3′) and Kmt2d_E4_R (5′-AGATGGCAACTCAAAGCGCTGC-3′). The geometric mean of the housekeeping genes *Actb* (5′-GGCCAACCGTGAAAAGATGA-3′ and 5′-CACAGCCTGGATGGCTACGT-3′) and *Hprt* (5′-TCAGTCAACGGGGGACATAAA-3′ and 5′-GGGGCTGTACTGCTTAACCAG-3′) was used. Data processing was performed with CFXmanager software (Bio-Rad).

#### Western blotting

Proteins were isolated from 1 mm of the spleen tissue, homogenized with traditional 1× Laemmli buffer and heated at 95°C for 5 min. To ensure comparable loading, proteins were isolated from equal amounts of starting material and processed under identical conditions. KMT2D protein levels and H3K4 methylation quantification was performed by western blotting with 4-20% Mini-PROTEAN^®^ TGX™ Precast Protein Gels (Bio-Rad, 4561093) and overnight transfer on PVDF membrane. Blocking was performed by 5% bovine serum albumin (BSA) in Tris-buffered saline (TBS) with 0.01% Tween for ∼4-6 h at room temperature. Primary antibody staining was conducted overnight at 4°C with the following antibodies: 1:1000 anti-KMT2D (MerckMillipore, ABE1867) with 1:1000 anti-vinculin (Sigma-Aldrich, V9131) as control; or 1:1000 H3K4me1 (Abcam, ab8895) or 1:1000 anti-H3K4me3 (Abcam, ab8580) with 1:3000 anti-H3 (Abcam, ab24834) as control. Secondary antibody staining was performed with IRDye^®^ 800CW Donkey anti-Rabbit IgG (H+L) (LiCor, 926-32213) and IRDye^®^ 680RD Donkey anti-Mouse IgG (H+L) (LiCor, 926-68072). Imaging was performed using Odyssey CLx (LiCor).

### Mouse phenotyping

#### Growth

The body weight of each animal was measured weekly from 4 to 8 weeks postnatally. Analyses of skull anomalies were conducted by microCT imaging performed on 6-month-old euthanized mice. The microCT system and pre-clinical optical imaging from MILabs (Houten, Netherlands) were used with the following settings: 50 kV, 75 ms exposure time and current of 0.21 μA, using a full-body scan. Reconstruction was performed with the MILabs software (v. 13.14) for conversion into NIfTI-1 data format file at voxel sizes of 80 μm. The files were then processed in 3D Slicer software (v. 5.6.2) (Fedorov et al., 2012) to add a set of 16 anatomical landmarks that were digitized on each specimen. Coordinates for landmarks for each specimen were imported into Rstudio for geometric morphometric analysis using the geomorph package (v. 4.1.0). Only one missing landmark was estimated using Thin-Plate Spline (TPS) interpolation. To isolate shape variation from scale, orientation and position, a generalized Procrustes analysis (GPA) was performed on the 3D coordinates. For visualization purposes and specific profile analyses, 3D coordinates were projected into 2D space, followed by a GPA to isolate shape variation from scale, orientation and position, and to optimize the 2D configuration. To test morphological differences, a Procrustes ANOVA was conducted using the procD.lm function with 999 permutations. Shape variation was visualized using PCA. Convex hulls were calculated for each group to illustrate morphospace occupation. To further explore group separation, a linear discriminant analysis with cross-validation was performed on the first five principal components (covering 93.7% of total variance), and a confusion matrix was generated to assess classification accuracy. Allometry, the relationship between shape and size, was analyzed by regressing shape scores against log-transformed centroid size, and regression slopes were calculated. Mean shape configurations were computed using the mshape function. Morphological differences were visualized through overlaid wireframes, with a magnification factor of 2 applied to highlight subtle shape changes. Long bones were isolated by dissection and measured at 3-4 months of age.

#### IgA blood serum levels

We assessed IgA levels in mouse blood serum samples using enzyme-linked immunosorbent assay (ELISA). Submandibular blood collection was performed on 12-week-old mice to obtain serum. After collection, blood samples were allowed to coagulate at room temperature for 30 min, then centrifuged at 1700 ***g*** for 10 min to separate the serum from the pellet. The assay was conducted according to the manufacturer's protocol for the Mouse IgA Uncoated ELISA Kit (Thermo Fisher Scientific, 88-50450-88). Corning™ Costar™ 9018 ELISA plates were coated with 100 μl capture antibody diluted in coating buffer/well. The plates were sealed and incubated overnight at 4°C. The following day, the wells were washed twice with 400 μl wash buffer/well. Thereafter, the wells were blocked with 250 μl blocking buffer at room temperature for 2 h. The wells were washed again as previously described. Standards and samples were prepared in twofold serial dilutions using assay buffer A (1×) and added to the wells. For blank wells, 100 μl assay buffer/well (1×) was used. The plates were sealed and incubated for 2 h at room temperature on a shaker at 400 rpm. The wells were washed, supplemented with 100 μl detection antibody/well, sealed and incubated on a shaker at 400 rpm at room temperature. After incubation, the washes were repeated a total of four times. Subsequently, 100 μl substrate solution/well was added to each well and incubated for 15 min. The reaction was stopped by adding an equal volume of stop solution. The absorbance of the plates was measured at 450 nm. One value was identified as a significant outlier according to Grubbs' test and was excluded from the analysis.

#### Behavioral testing

All behavior testing was conducted sequentially, starting at ∼8-10 weeks of age with the open field test, in which mice were left to explore the open field (50×50 cm) for 10 min and their behavior was tracked. Next, we performed the Y-maze test according to a published protocol (Prieur and Jadavji, 2019). Briefly, mice were placed in a three-armed maze with one arm blocked for 5 min to explore, followed by a trial with all arms available to explore. Measurements were made for the novel arm entries and total time spent in the arm, and calculations were performed for the spontaneous alternations (% spontaneous alternation=alternations/maximum alternations). The final behavior test conducted was the Morris water maze test, with the same setup as previously described (Bjornsson et al., 2014). A 150 cm diameter tank was filled with water that was allowed to equilibrate to room temperature, and nontoxic white paint was added. Mice were trained with 60 s trials, first with visible platform testing at four trials/day for 3 consecutive days. This was followed by a 5-day training period with four trials/day with a hidden platform. On the final day, the platform was removed, and the animal was allowed to swim for a total of 90 s. All tests were video recorded, measured and analyzed with Noldus equipment (Ethovision, version 14.0).

#### Immunofluorescence staining

After all behavior testing had been conducted, animals were injected with EdU over 7 consecutive days (50 μg/g/day), then euthanized with a ketamine (16 mg/ml) and xylazine (4 mg/ml) mixture and perfused with 4% paraformaldehyde (PFA), 24 h after the last injection. Brains were fixed with 4% PFA for 24 h followed by a serial sucrose gradient (10% to 20% to 30%). Brains were then embedded in optimal cutting temperature (OCT) compound (Tissue-Tek^®^ O.C.T., Sakura) and cryosectioned. On the first day of immunostaining, tissue sections were washed twice with TBS-T [TBS (LiCor, 927-60001) containing 0.025% Triton X-100 (Sigma-Aldrich, T8787-100ML)] for 10 min each. Antigen retrieval was performed by immersing the sections in sodium citrate buffer (pH 6) at 95°C for 20 min. Following antigen retrieval, sections were washed with 200 μl of 3% BSA prepared in PBS (pH 7.4) and subsequently incubated for 20 min with 200 μl permeabilization solution (0.5% Triton X-100 in PBS). The sections were washed again with 3% BSA in PBS. Then, 100 μl Click-iT™ Plus reaction cocktail, prepared according to the manufacturer's protocol (Click-iT™ Plus EdU Imaging Kit; Thermo Fisher Scientific, C10637), was added to each sample and incubated at room temperature for 1 h. The sections were subsequently washed once with TBS-T. The sections were blocked with TBS-T containing 3% normal goat serum (NGS; Abcam, ab7481) for 1 h at room temperature. Primary antibody, rabbit anti-histone H3 (tri-methyl-K4; Abcam, ab8580), was added to the blocking solution at a 1:300 dilution. The sections were incubated in this solution for 1 h at room temperature, then overnight at 4°C. The sections were washed three times with TBS-T, then incubated with secondary antibodies, goat anti-mouse IgG H&L (Alexa Fluor^®^ 647; Abcam, ab150115) and goat anti-rabbit IgG H&L (Alexa Fluor^®^ 555; Abcam, ab150078), diluted 1:1000 in blocking solution. The sections were incubated in this solution overnight at 4°C. After incubation, the sections were washed twice with TBS-T and stained with 1× Hoechst^®^ 33342 solution (5 μg/ml; Click-iT™ Plus EdU Imaging Kit, Thermo Fisher Scientific, C10637) for 1 h at room temperature, protected from light. The sections were then washed two to three times with PBS, mounted using ProLong™ Gold Antifade Reagent (Thermo Fisher Scientific, P36930) and stored at 4°C until imaging. Imaging was performed using a confocal microscope Cicero (CrestOptics) and an EVOS Auto microscope (Thermo Fisher Scientific). Image preprocessing and quantification were conducted using ImageJ.

#### Histology staining

Intestinal Peyer's patches were counted as previously described (Pilarowski et al., 2020). Kidneys were dissected from *Kmt2d^+/R5230H^* animals harboring either one or two kidneys, as well as from *Kmt2d^+/+^* animals for histology comparison. Hematoxylin and Eosin (H&E) staining was performed according to the protocol setup at the Department of Pathology, Landspitali – the National University Hospital of Iceland. Images of H&E-stained slides were captured with the EVOS Auto microscope, and glomeruli were measured using ImageJ.

### Statistics

Plots were created and statistics were performed with GraphPad Prism (v.10.1.1), using either mixed-effects ANOVA or unpaired two-tailed Student's *t*-tests. *P*≤0.05 was considered statistically significant.

## Supplementary Material

10.1242/dmm.052775_sup1 Supplementary information
