# Supplementary material for: A patient-derived missense mouse model of Kabuki syndrome 1
Source: Dis Model Mech. 2026 Oct 14;19(10):dmm052775. doi: 10.1242/dmm.052775 (PMC13714867; doi:10.1242/dmm.052775)
Supplement: Supplementary file 1 [file dmm-19-052775-s1.pdf]

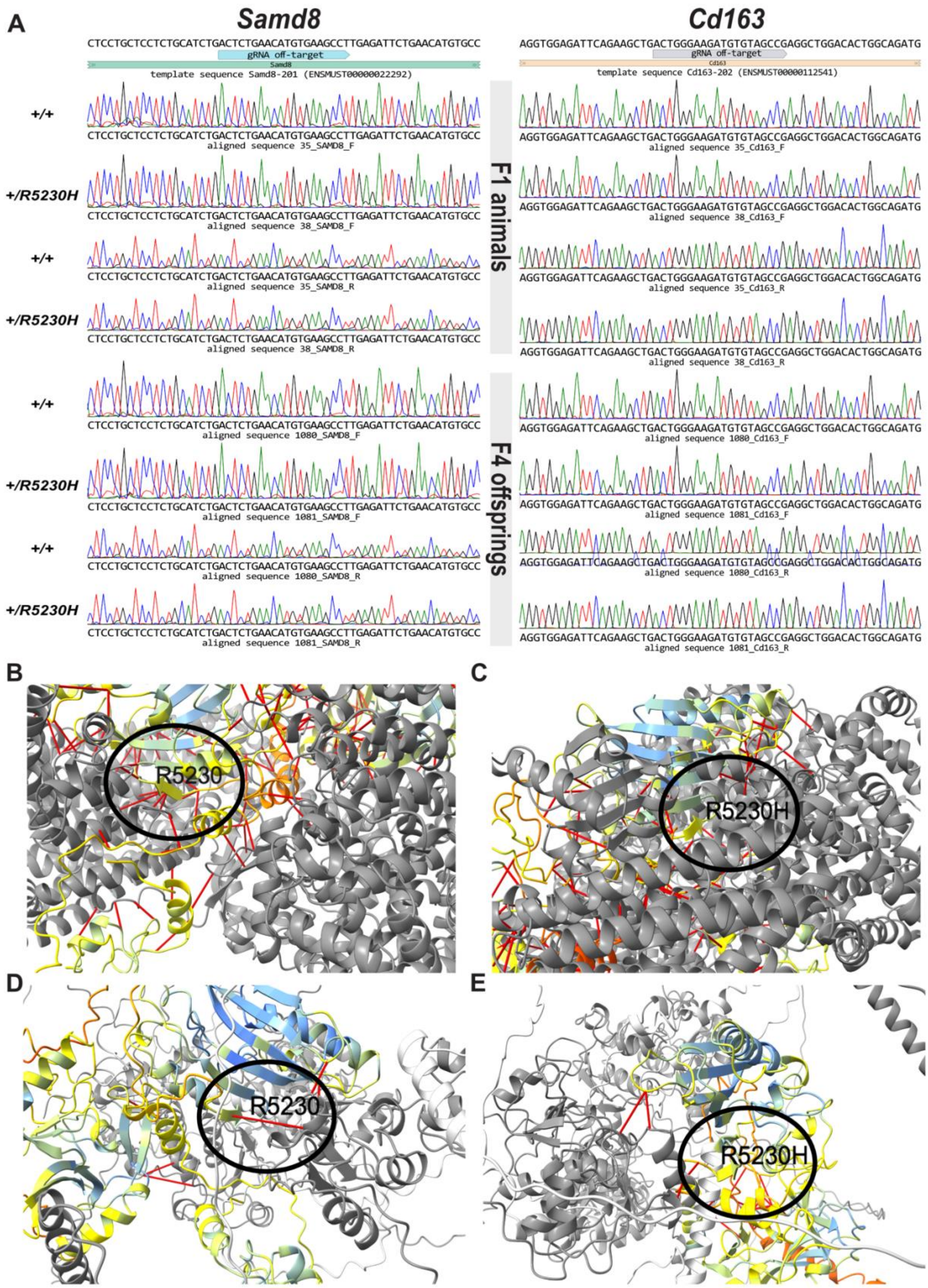

Disease Models & Mechanisms • Supplementary information

Fig. S1. Predicted off-target and AlphaFold prediction of interaction with COMPASS components. (A) Sanger sequencing results from CRISPR-Cas9 gRNA predicted off-target at *Samd8* (left) and *Cd163* (right) loci in F1 animals (top 4 tracks) and F4 offspring (bottom 4 tracks). AlphaFold3 prediction of part of KMT2D C-terminal starting at 100 amino acids downstream the FYRN domain. (B) Predicted interaction of NCOA6 colored in grey to R5230 or (C) R5230H KMT2D colored by pLDDT with circle showing interaction. (D) Predicted interaction of PAXI1 (grey) and its binding partner PGR1A (light grey) to R5230 or (E) R5230H KMT2D colored by pLDDT with circle showing interaction.

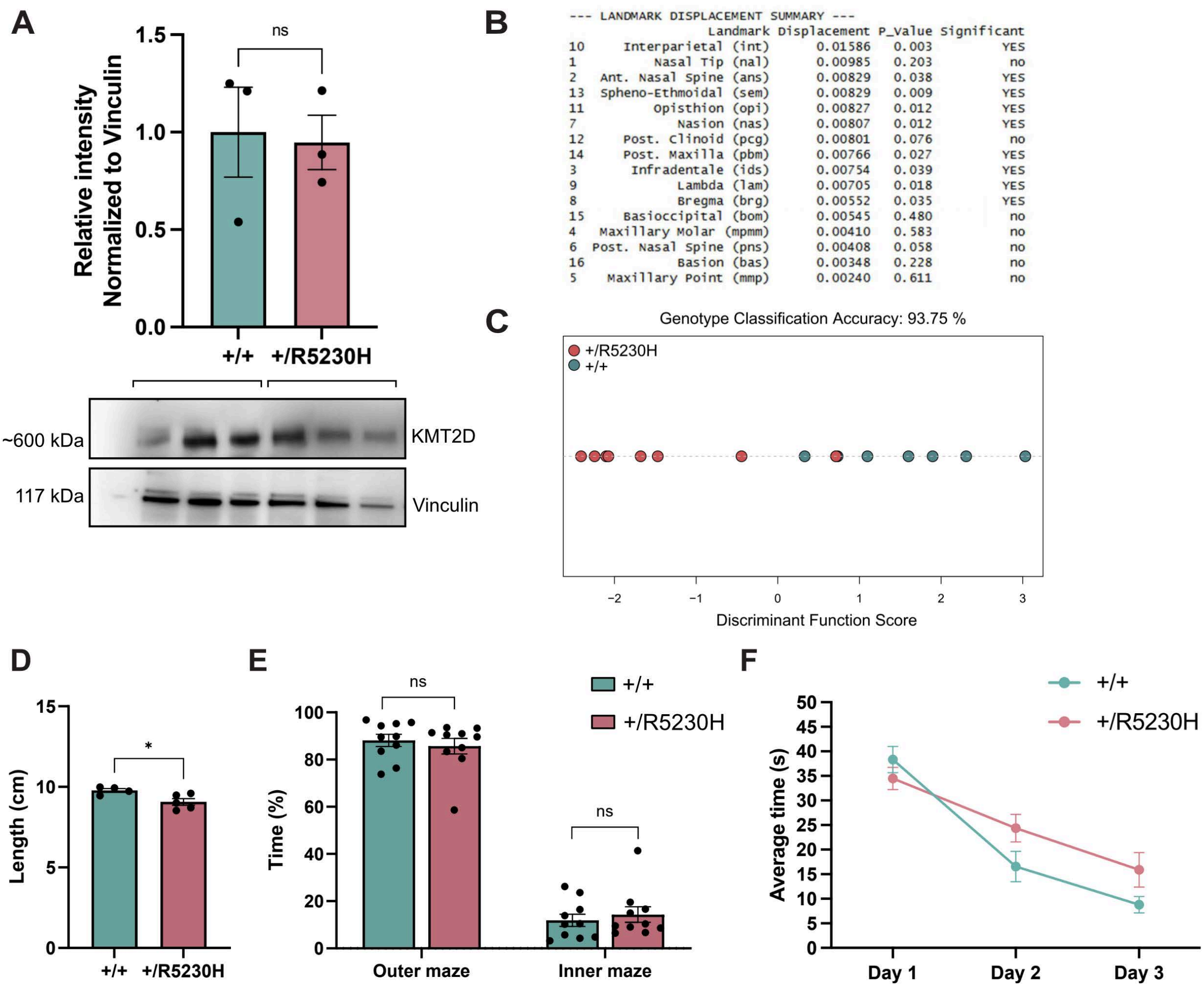

--- LANDMARK DISPLACEMENT SUMMARY ---

	Landmark	Displacement	P_Value	Significant
10	Interparietal (int)	0.01586	0.003	YES
1	Nasal Tip (nal)	0.00985	0.203	no
2	Ant. Nasal Spine (ans)	0.00829	0.038	YES
13	Spheno-Ethmoidal (sem)	0.00829	0.009	YES
11	Opisthion (opi)	0.00827	0.012	YES
7	Nasion (nas)	0.00807	0.012	YES
12	Post. Clinoid (pcg)	0.00801	0.076	no
14	Post. Maxilla (pbm)	0.00766	0.027	YES
3	Infradentale (ids)	0.00754	0.039	YES
9	Lambda (lam)	0.00705	0.018	YES
8	Bregma (brg)	0.00552	0.035	YES
15	Basioccipital (bom)	0.00545	0.480	no
4	Maxillary Molar (mpmm)	0.00410	0.583	no
6	Post. Nasal Spine (pns)	0.00408	0.058	no
16	Basion (bas)	0.00348	0.228	no
5	Maxillary Point (mmp)	0.00240	0.611	no

Fig. S2. Phenotypes consistent across mouse models. (A) Western blot from isolated mesenchymal stem cells (MSCs) and quantification of KMT2D with vinculin as control. (B) A tabular summary of the p-values and displacement along the craniofacial axis for each of the 16 landmarks. The landmarks are ranked from the most displaced to the least. (C) Linear Discriminant Analysis (LDA) of specimen morphology based on genotype using the first five principal components. (D) Body length measured in cm. (E) Open field test showing percentage of time spent in outer vs inner maze. (F) Morris water maze visible platform test across three training days with averages of 4 trials per day (n=10).

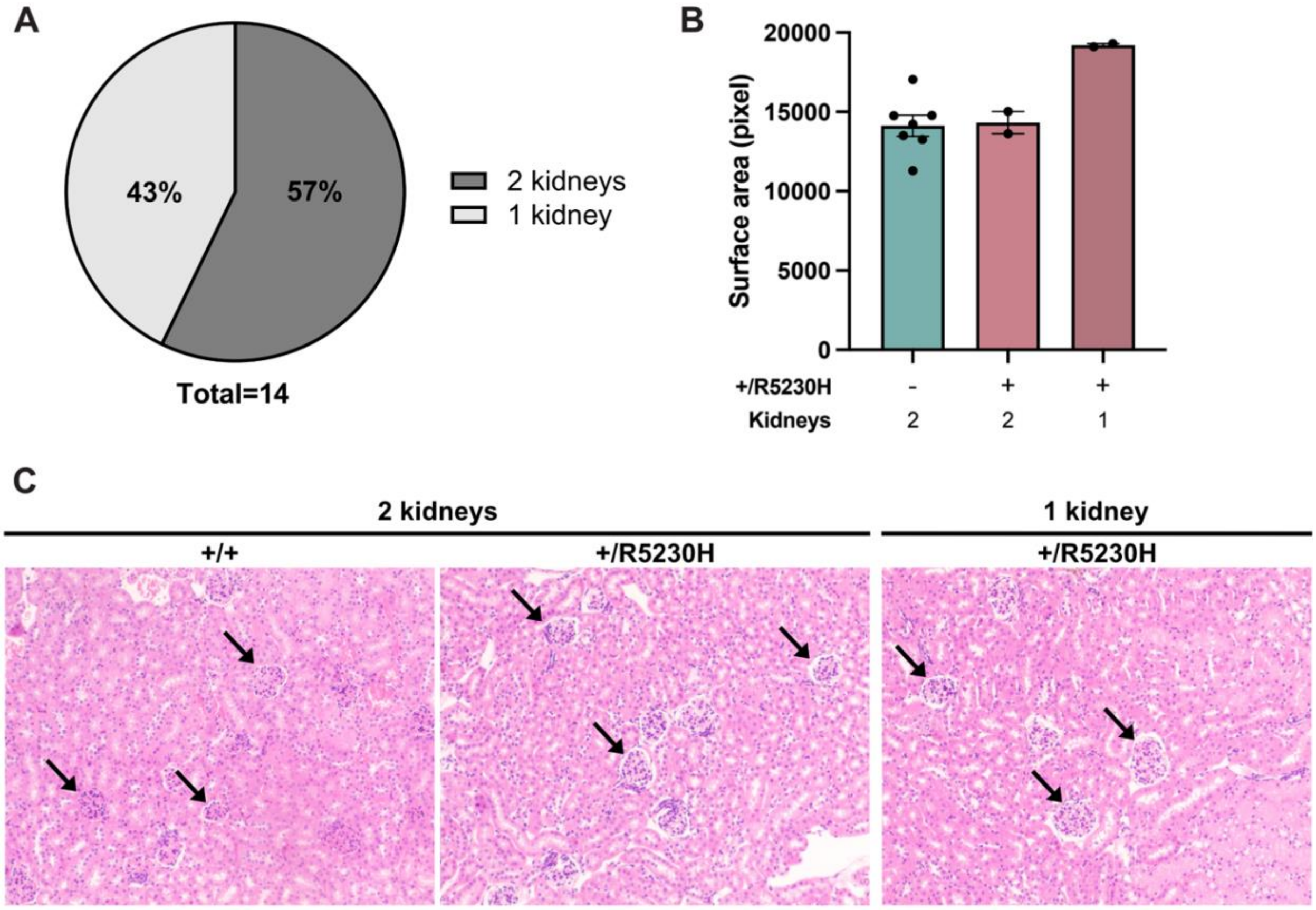

Fig. S3. Novel kidney phenotype found in *Kmt2d*$^{+/R5230H}$ mice. (A) Number of kidneys observed in R5230H mice with number of animals shown as percentage. (B) Average glomeruli size quantification (glomeruli n=7-12 per animal) and (C) representative images of H&E staining of kidneys from *Kmt2d*$^{+/+}$ (n=7) and *Kmt2d*$^{+/R5230H}$ mice with one (n=2) or two (n=2) kidneys. Data are represented as mean ± SEM.

Disease Models & Mechanisms • Supplementary information
